# Genetic Variants in Metabolic Pathways and Their Role in Cardiometabolic Risk: An Observational Study of >4000 Individuals

**DOI:** 10.3390/biomedicines13081791

**Published:** 2025-07-22

**Authors:** Angeliki Kapellou, Thanasis Fotis, Dimitrios Miltiadis Vrachnos, Effie Salata, Eleni Ntoumou, Sevastiani Papailia, Spiros Vittas

**Affiliations:** iDNA Laboratories, 7 Kavalieratou Taki, 14564 Kifisia, Greece; angie.kapellou@idna.gr (A.K.); athanasios.fotis@idna.gr (T.F.); dimitris.vrachnos@idna.gr (D.M.V.); effie.salata@idna.gr (E.S.); eleni.ntoumou@idna.gr (E.N.); sevastiani.papailia@idna.gr (S.P.)

**Keywords:** nutrigenetics, polymorphisms, cardiometabolic risk, obesity, type 2 diabetes

## Abstract

**Background/Objectives:** Obesity, a major risk factor for cardiometabolic traits, is influenced by both genetic and environmental factors. Genetic studies have identified multiple single-nucleotide polymorphisms (SNPs) associated with obesity and related traits. This study aimed to examine the association between genetic risk score (GRS) and obesity-associated traits, while incorporating SNPs with established gene–diet interactions to explore their potential role in precision nutrition (PN) strategies. **Methods**: A total of 4279 participants were stratified into low- and intermediate-/high-GRS groups based on 18 SNPs linked to obesity and cardiometabolic traits. This study followed a case–control design, where cases included individuals with overweight/obesity, T2DM-positive (+), or CVD-positive (+) individuals and controls, which comprised individuals free of these traits. Logistic regression area under the curve (AUC) models were used to assess the predictive power of the GRS and traditional risk factors on BMI, T2DM and CVD. **Results**: Individuals in the intermediate-/high-GRS group had higher odds of being overweight or obese (OR = 1.23, CI: 1.03–1.48, *p* = 0.02), presenting as T2DM+ (OR = 1.56, CI: 1.03–2.49, *p* = 0.03) and exhibiting CVD-related traits (OR = 1.56, CI: 1.25–1.95, *p* < 0.0001), compared to the low-GRS group. The GRS was the second most predictive factor after age for BMI (AUC = 0.515; 95% CI: 0.462–0.538). The GRS also demonstrated a predictive power of 0.528 (95% CI: 0.508–0.564) for CVD and 0.548 (95% CI: 0.440–0.605) for T2DM. **Conclusions**: This study supports the potential utility of the GRS in assessing obesity and cardiometabolic risk, while emphasizing the potential of PN approaches in modulating genetic susceptibility. Incorporating gene–diet interactions provides actionable insights for personalized dietary strategies. Future research should integrate multiple gene–diet and gene–gene interactions to enhance risk prediction and targeted interventions.

## 1. Introduction

Cardiometabolic traits, including type 2 diabetes mellitus (T2DM) and cardiovascular diseases (CVDs), are among the leading causes of morbidity and mortality worldwide [1,2]. These conditions share common metabolic pathways and risk factors, with obesity playing a central role in their pathophysiology [3,4,5]. The global prevalence of obesity has reached unprecedented levels, with an estimated 1 billion individuals worldwide affected as of 2025 [6]. Obesity is a major modifiable risk factor for T2DM, CVDs and other noncommunicable diseases (NCDs), contributing substantially to premature mortality [7,8]. These data underscore the critical importance of addressing obesity as a global public health priority.

Obesity is characterized by an abnormal or excessive accumulation of adipose tissue, leading to metabolic disturbances such as insulin resistance, chronic low-grade inflammation, dyslipidemia and hypertension, all of which contribute to cardiometabolic dysfunction [3,9,10]. Body mass index (BMI) is a widely used metric for classifying weight status, with a BMI of 25–29.9 kg/m^2^ indicating overweight and a BMI ≥30 kg/m^2^ defining obesity; both are associated with increased health risks [11,12]. Understanding the complex interactions between obesity and cardiometabolic traits is essential for developing effective prevention and intervention strategies.

The pathophysiology of obesity involves complex interactions among genetic, neurohormonal and environmental factors that disrupt appetite regulation, satiety and energy expenditure. While lifestyle and environmental exposures play a major role, genetic predisposition significantly influences individual susceptibility [13,14,15]. In rare cases, obesity arises from highly penetrant genetic mutations. Monogenic obesity, typically caused by leptin–melanocortin pathway gene defects (e.g., *LEP*, *LEPR*, *POMC*, *MC4R*), leads to severe early-onset hyperphagia and weight gain [16,17]. Similarly, syndromic obesity occurs as part of broader genetic syndromes with additional features, such as intellectual disability and dysmorphic traits, as observed in Prader–Willi and Bardet–Biedl syndromes [17].

More commonly, obesity is polygenic and multifactorial. Twin and family studies estimate that 40–75% of obesity risk is attributable to heritable factors [18,19,20]. Over the past decade, nearly 1000 Genome-Wide Association Studies (GWASs) have been published, identifying over 500 genetic loci and candidate single-nucleotide polymorphisms (SNPs) linked to BMI and obesity [21,22,23,24]. A well-known paradigm includes the *MC4R* (melanocortin 4 receptor) rs17782313 variant, which has been consistently reported to display an association with higher BMI in both children and adults [25,26,27]. Furthermore, risk allele C of this variant has been linked to cardiovascular risk factors such as insulin resistance, hypertension and inflammation [28,29,30,31,32]. Moreover, variants in the *FTO* (FTO alpha-ketoglutarate-dependent dioxygenase) gene have been linked to increased food intake and adiposity, while variants in *PPARG* (peroxisome proliferator-activated receptor gamma) are shown to influence appetite regulation, lipid metabolism and energy balance. These genetic variations, in combination with environmental and behavioral influences, contribute to interindividual differences in obesity susceptibility and metabolic outcomes [27].

Cardiometabolic traits, like T2DM, are also highly polygenic, involving multiple genes that regulate lipid metabolism, blood pressure and vascular function [33,34]. According to a recent review, almost 1700 studies have been conducted on ~450 phenotypes related to cardiovascular indicators and associated markers across the last 15 years [35]. These efforts have advanced knowledge of the genetic basis of these phenotypes, contributing to a deeper understanding of cardiometabolic biology and pathophysiology. Among the identified genetic variants, polymorphisms in *APOE* (apolipoprotein E) and *TCF7L2* (transcription factor 7 like 2) genes have been strongly linked to insulin resistance, dyslipidemia and atherosclerosis in different populations, thereby increasing the risk of coronary artery disease and T2DM [36,37,38,39].

A more refined approach to quantifying genetic susceptibility involves the use of genetic risk scores (GRSs), which aggregate risk alleles from multiple SNPs. Unlike GWASs, which require large sample sizes to detect significant associations, the GRS can enhance risk prediction even in smaller cohorts, making it a powerful tool for assessing cardiometabolic predisposition [40,41,42]. While genetic predisposition influences obesity risk, lifestyle remains a key determinant. An obesogenic environment amplifies genetic susceptibility, but a healthy lifestyle can significantly counteract this effect [43]. Regular physical activity, balanced nutrition and weight management not only modulate genetic susceptibility to obesity, but may also lower the incidence of obesity-related diseases [44]. Therefore, individuals with high genetic susceptibility may benefit the most from individualized lifestyle changes and precision nutrition (PN) strategies, as they face the greatest risk of obesity and its associated complications.

Despite the growing body of research on genetic determinants of obesity and associated phenotypes, relatively few studies have explored the association between obesity-related GRS and cardiometabolic traits. Therefore, this study aims to compute a GRS based on obesity-related markers to investigate how genetic predisposition is associated with the development of obesity, T2DM and CVD in Greece. The findings will contribute to the growing evidence on the genetic basis of disease risk while underscoring the potential of gene-based interventions in advancing personalized prevention and treatment strategies for cardiometabolic health.

## 2. Materials and Methods

### 2.1. Study Population

Participants were selected from a large set of samples (n = 13,180) originally collected for commercial genetic testing purposes between January 2021 and December 2024. The study was conducted in accordance with the ethical principles outlined in the Declaration of Helsinki and its subsequent amendments. A standardized lifestyle questionnaire was introduced partway through the data collection period; therefore, only individuals sampled after its implementation had the opportunity to complete it. Inclusion criteria required participants to have both genetic data and a completed questionnaire, with written informed consent for the use of combined data in research.

**Figure 1:**
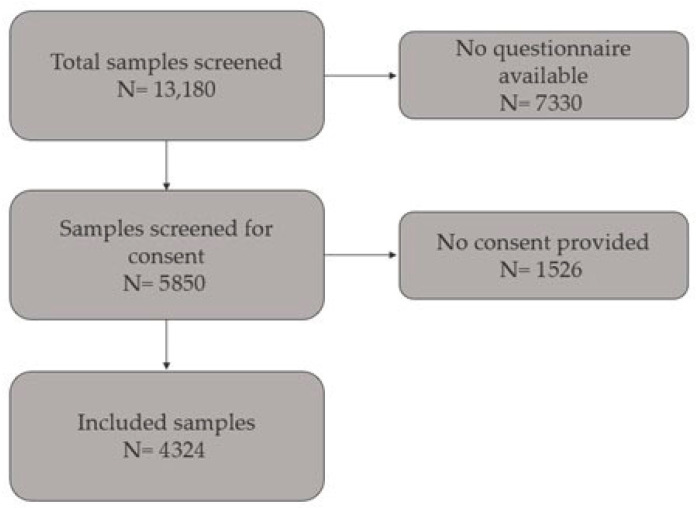
Flowchart for sample selection.

After excluding individuals who were sampled before the questionnaire was implemented or who did not complete it, a final cohort of 4324 participants was included in the analysis (Figure 1). The questionnaire was completed online and collected information on anthropometric measurements (weight and height), medical history, medication, and dietary supplement use.

### 2.2. Phenotypic Classification

Participants were classified into distinct groups based on BMI and the presence of cardiometabolic traits. BMI was used to categorize individuals as normal-weight (BMI < 25 kg/m^2^) or overweight/obese (BMI ≥ 25 kg/m^2^) in accordance with established clinical guidelines [45]. Individuals were also categorized as T2DM-positive (+) or T2DM-negative (−) based on self-reported medical history and/or the use of glucose-lowering medication, including insulin, metformin, or sulfonylureas. T2DM+ individuals were those with positive T2DM history and/or who were using at least one of the aforementioned medications, while those on GLP-1 receptor agonists or medications specifically prescribed for weight loss were categorized as T2DM-. The presence of cardiovascular-related traits was determined based on self-reported medical history, including conditions such as hypertension, coronary artery disease, stroke and dyslipidemia and/or associated medication. Participants were then categorized into CVD-positive (+) or CVD-negative (−) groups. This classification allowed for a structured evaluation of cardiometabolic risk factors in relation to genetic predisposition.

### 2.3. SNP Selection and Genotyping

Eighteen SNPs were selected based on their established associations with obesity and obesity-associated traits from GWASs [18,20,21,46] (Table 1). These SNPs were specifically selected for their demonstrated interactions with nutrients, as only such variants can provide actionable insights and effectively guide personalized nutrition recommendations [47,48].

Genotyping was performed using TaqMan Custom OpenArray Genotyping Assays from the QuantStudio™ 12 K Flex Real-Time PCR System (Applied Biosystems by Life Technologies, Carlsbad, CA, USA). The panel consists of 18 target SNPs (Table 1). The corresponding sets of primers and probes were included in the custom Openarray Genotyping plates purchased from ThermoFischer Scientific (Waltham, MA, USA). The genotyping method was performed following the manufacturer’s protocol. The temperature protocol (95 °C for 45″ and 60 °C for 45″ for 50 cycles) was followed in accordance with the manufacturer’s instructions. Negative controls did not include DNA and yielded no amplification signal. Raw data were collected from the instrument and analyzed with the QuantStudio 12 K Flex software v1.6 and the TaqMan Genotyper Software v1.7.1, via the method of allelic discrimination.

### 2.4. Statistical Analysis

Statistical analyses were conducted using R Programming software (version 4.0.3) and Python version 3.12 (statsmodel v0.14.4 package). As part of data quality control, BMI outliers were excluded via boxplot visualization [49]. The Shapiro–Wilk test was used to check the normality of the data. Descriptive analysis was used to present the general characteristics of participants by means ± standard deviation (SD) or medians ± interquartile range (IQR), depending on normality. Group differences were assessed using independent samples t-tests or Mann–Whitney U tests for continuous variables and Chi-square or Fisher’s exact tests for categorical variables.

Deviations from the Hardy–Weinberg Equilibrium (HWE) for each SNP were evaluated using the *χ*^2^ goodness-of-fit test. A GRS was derived from 18 SNPs identified through an extensive search of the literature, encompassing variants associated with obesity, CVD and T2DM. These SNPs were selected not only for their established links to cardiometabolic risk but also for their demonstrated gene–diet interactions, which are shown to influence the modulation of this risk. The GRS was computed using an additive model, where each risk allele contributed 0, 1, or 2 points, depending on individual genotype. The GRS scale ranged from 0 to 36, with each point corresponding to one risk allele. For standardization and enhanced interpretability, the scale was subsequently transformed to a range from 0 to 100. Higher scores indicate greater genetic susceptibility to higher BMI and cardiometabolic traits [50]. The score was also stratified into tertiles, ‘low’ (bottom third, ≤33.3), ‘intermediate’ (middle third, >33.3–66.6), and ‘high’ (top third, ≥66.7), and analyzed as categorical variables in statistical models. GRS group differences were assessed using independent samples t-tests or Mann–Whitney U tests for continuous variables and Chi-square or Fisher’s exact tests for categorical variables.

The area under the curve (AUC) was used to describe the discriminative ability of all available variables in predicting obesity and cardiometabolic traits. Logistic regression models were fitted for each variable, and the corresponding AUC scores were extracted. To estimate 95% confidence intervals (CIs), a bootstrap resampling method with 1000 iterations was applied, excluding missing values. Additionally, to minimize potential bias in AUC estimation due to random splitting of the dataset, a 10-fold cross-validation approach was implemented before bootstrapping. Statistical significance was set at *p* < 0.05.

## 3. Results

### 3.1. Participant Characteristics

A total of 45 participants were excluded due to extreme BMI values, identified as outliers through boxplot visualization applying Tukey’s outer fences (values < Q1 − 3 × IQR or > Q3 + 3 × IQR), which is appropriate for skewed distributions [49]. Therefore, a total of 4279 participants were included in this study, of whom 75.4% were female. The median age of participants was 41.04 ± 11.61 years (Table 2). Participants were stratified into low-GRS (n = 619) and intermediate-/high-GRS (n = 3660) groups, since only three participants were classified in the high-GRS group. The median GRS in the total sample was 41.67 ± 11.11, with significantly different distributions between the low-GRS and the intermediate-/high-GRS group, confirming the expected distinction between the two categories.

The mean BMI of the total sample was 29.09 ± 6.21 kg/m^2^, with no differences between the GRS groups. The proportions in terms of sex (males vs. females) and smoking status (yes vs. no) were similar across GRS groups. The median age did not differ significantly between groups, with the low-GRS group averaging 40.83 ± 11.73 years and the moderate-/high-GRS group averaging 41.08 ± 11.59 years.

**Figure 2:**
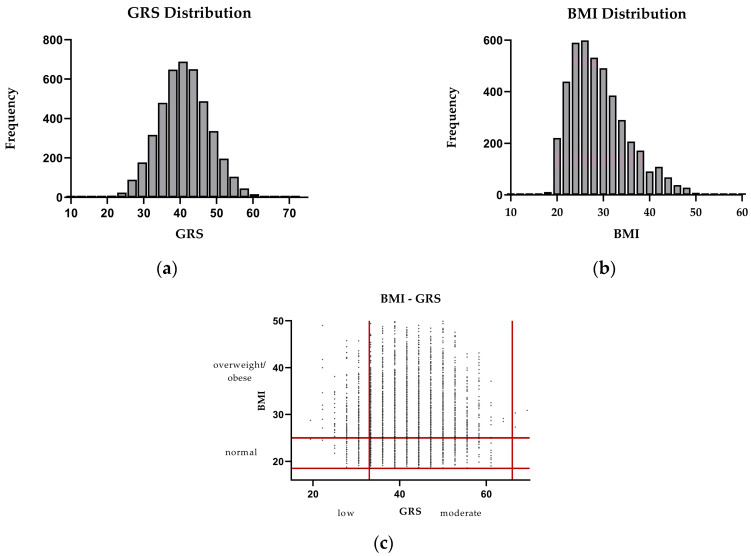
Distribution of genetic risk score (GRS) and body mass index (BMI) in study population. (**a**) Histogram displaying the frequency of individuals across different GRS values on a scale of 0-100; (**b**) histogram displaying the frequency of individuals across different BMI values; (**c**) scatterplot illustrating the distribution of participants based on GRS and BMI. Horizontal red lines represent the normal BMI range (18.5 to 24.9 kg/m^2^), while vertical red lines denote the boundaries for low and intermediate/high GRS.

The distribution of GRSs follows an approximately normal pattern, with most participants clustered around the median GRS value. In contrast, the BMI distribution is left-skewed, indicating that most individuals fall within the range of 20 to 35 and a smaller proportion exhibit higher BMI values. Based on the scatterplot, most participants fall within the overweight/obese–high-GRS categories (Figure 2).

### 3.2. Associations Between GRS and Obesity, and Between CVD and T2DM

**Figure 3:**
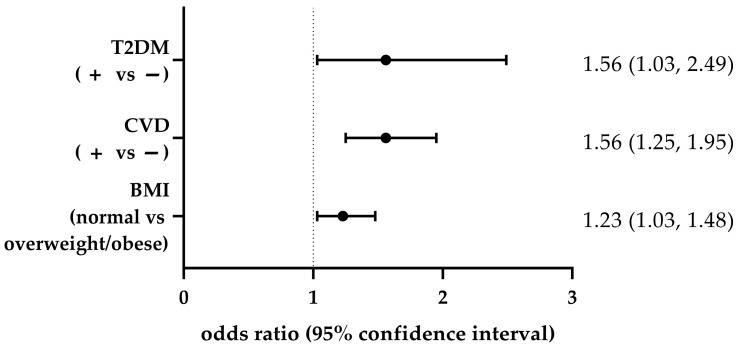
Association between genetic risk score (GRS) and health-related outcomes. The forest plot displays the odds ratios (ORs) and corresponding 95% confidence intervals (CIs) for the association between GRS and type 2 diabetes (T2DM), cardiovascular disease (CVD) and body mass index (BMI). The vertical line at OR = 1 represents the null hypothesis, indicating no association.

Individuals in the intermediate-/high-GRS group had a higher prevalence of overweight/obesity (1055 cases) compared to the low-GRS group (206 cases), with a 23% increased likelihood of being overweight or obese (OR = 1.23, 95% CI: 1.03–1.48, *p* = 0.02). A similar pattern was observed for T2DM, where the intermediate-/high-GRS group had 209 T2DM+ individuals compared to 23 T2DM+ in the low-GRS group, corresponding to a 56% higher risk (OR = 1.56, 95% CI: 1.03–2.49, *p* = 0.03). Likewise, a significantly greater number of CVD+ individuals was observed in the intermediate-/high-GRS group (914 individuals) compared to the low-GRS group (109 individuals), indicating a 56% higher risk of being CVD+ (OR = 1.56, 95% CI: 1.25–1.95, *p* <0.0001) in the high-GRS group (Figure 3).

### 3.3. Discriminative Ability of GRS

When assessing the discriminative ability of the GRS for BMI, the dataset exhibited a significant class imbalance. To mitigate potential bias and ensure robust metric estimation, several corrective measures were applied. When categorizing individuals as either normal-weight (n = 1212) or overweight/obese (n = 2912), an imbalance was particularly evident in the distribution of age, which in turn influenced the BMI AUC score. To address this, the overweight/obese category was resampled by approximating the Gaussian distribution of the normal-weight category (Figure S1).

**Figure 4:**
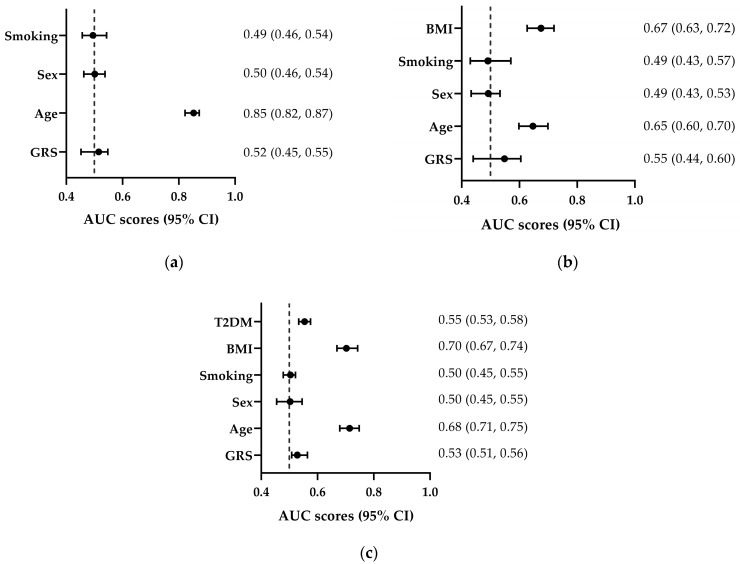
Discriminative ability (AUC) of predictors for BMI, type 2 diabetes (T2DM) and cardiovascular disease (CVD). Forest plot displaying AUC values for predictors of (**a**) BMI; (**b**) T2DM; and (**c**) CVD. Error bars indicate 95% confidence intervals. Vertical lines indicate AUC values of 0.50, below which there is no predictive ability, while higher values indicate better discrimination.

GRS demonstrated a modest predictive ability for BMI (AUC = 0.515, 95% CI: 0.452–0.548), outperforming sex (AUC = 0.501, 95% CI: 0.462–0.538) and smoking (AUC = 0.495, 95% CI: 0.457–0.543), while age had the strongest discriminative power (AUC = 0.853, 95% CI: 0.822–0.873). For CVD and T2DM classification, balanced datasets were created by randomly downsampling the overrepresented categories (CVD- and T2DM-). In CVD classification, GRS (AUC = 0.528, 95% CI: 0.508–0.564) ranked fourth in predictive impact, following age (AUC = 0.714, 95% CI: 0.679–0.748), BMI (AUC = 0.703, 95% CI: 0.669–0.743), and T2DM (AUC = 0.554, 95% CI: 0.533–0.575). In T2DM classification, GRS (AUC = 0.548, 95% CI: 0.440–0.605) was the third most predictive factor, following BMI (AUC = 0.675, 95% CI: 0.627–0.720) and age (AUC = 0.675, 95% CI: 0.627–0.720) (Figure 4).

## 4. Discussion

This study aimed to investigate the association between genetic predisposition, obesity and cardiometabolic traits, incorporating variants with demonstrated gene–diet interactions. The findings confirm that, in addition to lifestyle and environmental influences, a higher GRS is associated with an increased risk of obesity, T2DM and CVD, reinforcing the role of genetic susceptibility in cardiometabolic disease. These results highlight the potential of PN approaches that integrate genetic screening alongside conventional markers, providing a framework for personalized interventions tailored to individual genetic profile.

The predictive power of obesity polygenic risk scores (PRSs) has improved significantly due to advancements in GWASs over the past decades [51]. Most GWASs have used BMI as a proxy for obesity, as it is practical for large-scale studies. Since the identification of the first obesity-associated locus (*FTO*) in 2007 [52], GWAS sample sizes have grown exponentially, now nearing one million participants [46]. This expansion has led to the discovery of over 500 genomic loci linked to BMI, implicating genes involved in metabolism, adipose tissue function and neuronal processes of satiety [24,46].

A study of 33,511 European adults developed an obesity PRS based on 97 BMI-associated variants, demonstrating strong associations with abnormal weight gain (OR = 1.71, 95% CI: 1.38–2.13), obesity (OR = 2.08, 95% CI: 1.83–2.36), morbid obesity (OR = 2.88, 95% CI: 2.40–3.45) and bariatric surgery (OR = 3.01, 95% CI: 2.19–4.14). Furthermore, each SD increment in PRS increased obesity risk by 1.26-fold (95% CI: 1.22–1.29). Some associations were attenuated after adjusting for BMI, indicating that the genetic risk is likely mediated through obesity per se [53].

Despite a smaller sample size, this study also found a significant association between genetic risk and BMI, with an intermediate-/high-GRS demonstrating a 1.23-fold increased likelihood of being overweight or obese (95% CI: 1.03–1.48). However, unlike previous studies based on hundreds of thousands of individuals, this study had a substantially smaller sample, limiting the power to detect stronger genetic associations. Furthermore, while large GWAS-based PRS models incorporate hundreds of SNPs, this study used fewer variants, prioritizing those with known dietary interactions. Despite these differences, this distinction makes the present findings particularly relevant for PN, as they highlight genetic risks that may be modifiable through dietary interventions.

The present study aligns with prior research demonstrating the polygenic nature of T2DM and the predictive value of GRS in assessing disease susceptibility. A study partitioned T2DM genetic variants into five metabolic pathways, including obesity, lipodystrophic insulin resistance, liver/lipid metabolism, hepatic glucose metabolism and β-cell dysfunction. The findings showed that distinct genetic clusters contribute to T2DM risk through different mechanisms, with some associated with lower BMI, while others, such as the obesity PRS, being linked to higher BMI (Beta = 0.08, *p* = 8.0 × 10^−33^) [54].

A study assessing weighted (wGRS) and unweighted (uGRS) genetic risk scores found that individuals with higher uGRS values had twice the risk of developing T2DM (OR = 2.00, CI: 1.72–2.32, *p* < 0.0001), with similar results replicated in two independent T2DM cohorts. Additionally, the wGRS further improved risk estimations (*p* < 0.000001), underscoring the robustness of wGRS in predicting T2DM susceptibility. This study also assessed diabetes-related complications, demonstrating that GRS was also predictive of neuropathy (*p* < 0.0001), nephropathy (*p* < 0.005) and leg ischemia (*p* < 0.0005) [55].

Similarly, the present study revealed a 56% increased likelihood of any T2DM-related trait in the intermediate-/high-GRS group (95% CI: 1.03–2.49), reinforcing the genetic contribution to diabetes susceptibility. However, key differences exist between this study and previous research. Unlike prior studies that incorporated a weighted GRS, an unweighted score we used herein, which may have influenced the risk estimates. Additionally, while earlier research stratified genetic risk into metabolic subtypes, this study did not account for mechanistic heterogeneity within T2DM risk variants. Instead, SNPs with demonstrated gene–diet interactions were selected, emphasizing the modifiable nature of genetic susceptibility, an aspect not explored in other studies.

Another distinction is in SNP selection—while some variants were shared between studies, the present analysis specifically included SNPs that have demonstrated interactions with diet, supporting their relevance for PN applications. Moreover, the present dataset relied on self-reported data, which, albeit valuable, may have introduced potential reporting biases. Finally, T2DM+ individuals in this study were not strictly diabetic, which may have influenced associations with genetic risk. Despite these limitations, the present investigation extends the existing literature by highlighting modifiable genetic risks and the potential for PN approaches in T2DM treatment and prevention.

There is substantial evidence linking genetics to CVD. Previous research has demonstrated that individuals in the top 95th percentile of a coronary artery disease (CAD) PRS, based on hundreds of loci, have three-fold increased odds of developing CAD, even in the absence of severe hypercholesterolemia [56,57]. Similarly, another study found that those in the top 10th percentile of PRS had 5.7-fold greater odds (95% CI: 2.60–13.95) of developing early-onset atrial fibrillation compared to those in the bottom 90th percentile [58].

Further evidence from UK Biobank supports a shared genetic basis among multiple CVD traits. A PRS constructed from 300 CAD-associated variants was significantly associated with various CVD risk factors and outcomes, including hypercholesterolemia (OR = 1.27; 95% CI: 1.26–1.29), hypertension (OR = 1.11; 95% CI: 1.10–1.12), peripheral arterial disease (OR = 1.28; 95% CI: 1.23–1.32), and atrial fibrillation (OR = 1.08; 95% CI: 1.05–1.10). These findings suggest that CVD traits share genetic underpinnings, with potential overlapping pathophysiological mechanisms [59].

In line with these findings, the present study also identified a 56% increased risk of presenting any CVD trait (OR = 1.56, 95% CI: 1.25–1.95) in individuals with a high GRS. However, a key distinction is that while previous studies incorporated hundreds of SNPs, the GRS of this study was based on a more selective set of variants chosen specifically for their known interactions with dietary factors. This makes the present approach particularly relevant for dietary interventions to modify CVD risk. Additionally, unlike studies that examined individual cardiovascular outcomes separately, a broader definition of CVD traits was used in this investigation, classifying individuals with any cardiovascular trait—including hypercholesterolemia or hypertension—as cases. This approach captures a more comprehensive spectrum of CVD risk and aligns with the growing recognition that genetic susceptibility extends beyond single-trait endpoints. Therefore, the present findings reinforce the concept of a shared genetic background among CVD traits, while also demonstrating the potential for dietary modifications to modulate risk in genetically predisposed individuals. However, it should be acknowledged that cardiovascular risk can also be indirectly assessed from an early age using established clinical measures, such as anthropometric indices and baseline laboratory tests, which remain essential tools in routine risk stratification and complement genetic approaches [60].

The findings of this study align with previous research that underscores the predictive value of GRS in assessing obesity risk. For example, a study within the Healthy Lifestyle in Europe by Nutrition in Adolescence (HELENA) cohort used a GRS of 21 obesity-associated SNPs and reported an AUC of 0.723, supporting the predictive ability of GRS for overweight and obesity classification [59]. Other studies using similar methodologies have reported an AUC of 0.701 [61] and 0.573 [62] in adults. In the present study, the GRS demonstrated a higher predictive ability (an AUC of 0.515) compared to traditional factors including sex (AUC = 0.501) and smoking (AUC = 0.495) but failed to surpass the predictive power of age (AUC = 0.853).

When categorizing the data based on T2DM and CVD traits, balanced datasets were created by random sampling from the overrepresented categories (T2DM- and CVD-). In T2DM classification, GRS (AUC = 0.548) ranked third in predictive power, after BMI (AUC = 0.675) and age (AUC = 0.675). Similarly, in CVD classification, GRS (AUC = 0.528) proved to be the fourth most impactful variable, following age (AUC = 0.714), BMI (AUC = 0.703) and T2DM (AUC = 0.554). These findings highlight the modest discriminative ability of GRS compared to traditional risk factors, underscoring the dominant roles of age and BMI in predicting cardiometabolic traits. Nevertheless, it is important to note that in this sample, age may have acted as a confounder, as individuals with lower BMI were significantly younger than those with higher BMI (Figure S1), which may have led to an overestimation of the discriminative ability of age. Additionally, the majority of individuals were classified as overweight or obese. Therefore, the present results contribute to the growing body of evidence suggesting that GRS can be a valuable tool for improving obesity risk prediction when combined with traditional risk factors, in some cases outperforming them.

Supporting this interpretation, Mendelian randomization (MR) studies have demonstrated that BMI exerts a causal effect on the development of T2DM and CVDs, suggesting that genetic predisposition to higher BMI may directly contribute to the increased risk of cardiometabolic diseases [63,64,65]. Indeed, a MR study using UK Biobank data constructed a GRS of 93 SNPs and found that genetically predicted higher BMI was significantly associated with hypertension (OR = 1.64, *p* = 1.1 × 10^−19^), coronary heart disease (OR = 1.35, *p* = 0.007) and T2DM (OR = 2.53, *p* = 1.5 × 10^−17^) [63]. Additionally, muti-variable MR indicates that 1 standard unit increase in BMI increases coronary artery risk by 1.49-fold. Approximately 70% of the effects of increased BMI on coronary artery disease are mediated by systolic blood pressure (~27% attributed), T2DM (~41% attributed), lipids (~3%) and smoking (~6%). Nonetheless, after adjusting for these factors, a standard deviation increase in BMI was still associated with 1.14-fold increase in coronary artery disease risk [65].

These results reinforce the role of genetic predisposition in obesity-related disease risk while emphasizing the causal link between BMI and cardiometabolic outcomes. Consistently, the present study demonstrated that individuals in the intermediate-/high-GRS group exhibited a significantly increased likelihood of being overweight or obese (23%) and presenting with any T2DM (56%) and CVD (56%) trait. While large-scale MR studies provide robust evidence for causal inference, targeted GRS models such as the one utilized in this study, which integrate gene–diet interactions, may enhance PN strategies, facilitating a more personalized approach to modulating genetic susceptibility.

A notable strength of this study is the relatively homogenous Greek ancestry of participants, which likely reduced population stratification and strengthened internal validity. However, this same homogeneity may limit the generalizability of the findings to more genetically diverse or non-European populations. Nevertheless, prior studies have shown that the Greek population is representative of Southern European genetic variation [66,67], suggesting that our findings may still be broadly relevant within European ancestry contexts. A key strength of this study is the large sample size, allowing for robust statistical analyses of GRS and cardiometabolic risk factors. Additionally, the selection of 18 SNPs with demonstrated gene–diet interactions enhances the relevance of these findings for PN applications by providing usable information on dietary strategies tailored to genetic risk. These loci not only contribute to obesity and cardiometabolic risk, but also present opportunities for targeted dietary interventions to modulate genetic susceptibility.

Nonetheless, some limitations should be acknowledged. First, the observational design of this study precludes causal inferences. Second, lifestyle factors such as physical activity and dietary intake were not assessed, potentially influencing the observed associations. Third, gene–gene interactions were not explored, which could improve future predictive models. Moreover, the sample’s representativeness may be limited, as age-related confounders and the predominance of overweight and obese participants—likely more health-conscious or interested in weight management—may have biased associations and reduced the generalizability of results. Finally, obesity and cardiometabolic traits are multifactorial conditions, and herein, a limited set of traditional risk factors was considered.

## 5. Conclusions

The present findings suggest that a GRS incorporating variants associated with obesity and cardiometabolic traits that have demonstrated established gene–diet interactions can help predict the likelihood of these conditions. This insight reinforces the potential of PN as a targeted approach to modulating genetic risk through personalized dietary and lifestyle interventions. Furthermore, these findings provide a foundation for future nutrigenetics research to assess the effectiveness of PN strategies in the prevention and management of cardiometabolic diseases, ultimately advancing the integration of genetics into personalized healthcare.

## Figures and Tables

**Table 1:** Overview of the 18 SNPs included in the GRS.

Gene	rs Number
*ACE*	rs4343
*ADH1C*	rs283411
*ADORA2A*	rs5751876
*ADRB2*	rs1042713
*APOC3*	rs5128
*APOE*	rs429358
*APOE*	rs7412
*CLOCK*	rs1801260
*CYP1A2*	rs762551
*FADS2*	rs174570
*FTO*	rs9930506
*FTO*	rs9939609
*LIPC*	rs1800588
*MC4R*	rs17782313
*MCM6*	rs4988235
*SLC2A2*	rs5400
*TCF7L2*	rs12255372
*TCF7L2*	rs7903146

Nearest gene of located single-nucleotide polymorphism (SNP); SNP rs number.

**Table 2:** Main characteristics of study participants.

	Total (n = 4279)	Low GRS (n = 619)	Intermediate/High GRS (n = 3660)	*p*
Age ^1^, years	41.04 ± 11.61	39.60 ± 17.20	39.90 ± 17.12	0.53
Sex, M/F (%)	1054 (24.6)/ 3225 (75.4)	139 (22.5)/ 480 (77.5)	915 (25.0)/ 2745 (75.0)	0.19
BMI ^1^, kg/m^2^	29.09 ± 6.21	28.67 ± 6.09	29.16 ± 6.22	0.06
BMI categories, normal/overweight/obese (%)	1261 (29.5)/3018 (70.5)	206 (33.3)/413 (66.7)	1055 (28.8)/2065 (71.2)	0.02
T2DM, yes/no (%)	232 (5.4)/4047 (94.6)	23 (3.7)/596 (96.3)	209 (5.7)/3451 (94.3)	0.04
CVD, yes/no (%)	1023 (23.9)/3256 (76.1)	109 (17.6)/510 (82.4)	914 (25.0)/2746 (75.0)	<0.001
Smoking status, yes/no (%)	1028 (24.8)/ 3096 (75.1)	162 (27.2)/ 433 (72.8)	866 (24.5)/ 2663 (75.5)	0.16
Genetic Risk Score ^1^ (GRS)	41.67 ± 11.11	33.33 ± 2.78	44.40 ± 8.33	<0.001

^1^ The values are presented as median ± IQR and the Mann–Whitney–Wilcoxon test was performed to observe differences between GRS groups; the Chi-square test was performed to observe differences in GRS groups between the BMI, T2DM, CVD and smoking status categories.

## Data Availability

The data presented in this study are available upon request from the corresponding author. The data are not publicly available due to restrictions imposed by the data owner, as they are the proprietary property of iDNA Laboratories S.A., Private Diagnostic Laboratories.

## Supplementary Materials

The following supporting information can be downloaded at https://www.mdpi.com/article/10.3390/biomedicines13081791/s1: Figure S1: Age distribution of study sample (a) before sampling and (b) after sampling to balance age between BMI categories.

## Abbreviations

The following abbreviations are used in this manuscript:

*ACE*	Angiotensin I Converting Enzyme
*ADH1C*	Alcohol Dehydrogenase 1C (Class I), Gamma Polypeptide
*ADORA2A*	Adenosine A2a Receptor
*ADRB2*	Adrenoceptor Beta 2
*APOC3*	Apolipoprotein C3
*APOE*	Apolipoprotein E
*AUC*	Area Under the Curve
*BMI*	Body Mass Index
*CLOCK*	Clock Circadian Regulator
*CVD*	Cardiovascular Disease
*CYP1A2*	Cytochrome P450 Family 1 Subfamily A Member 2
*FADS2*	Fatty Acid Desaturase 2
*FTO*	FTO alpha-ketoglutarate dependent dioxygenase
*GRS*	Genetic Risk Score
*GWAS*	Genome-Wide Association Study
*MR*	Mendelian Randomization
*LEP*	Leptin
*LEPR*	Leptin Receptor
*LIPC*	Hepatic Lipase C
*MC4R*	Melanocortin 4 Receptor
*MCM6*	Minichromosome Maintenance Complex Component 6
*PN*	Precision Nutrition
*POMC*	Pro-Opiomelanocortin
*SLC2A2*	Solute Carrier Family 2 Member 2 (Glucose Transporter Type 2)
*T2DM*	Type 2 Diabetes Mellitus
*TCF7L2*	Transcription Factor 7 Like 2

## References

[1] Wong N.D., Sattar N. (2023). Cardiovascular risk in diabetes mellitus: Epidemiology, assessment and prevention. Nat. Rev. Cardiol..

[2] Chakraborty S., Verma A., Garg R., Singh J., Verma H. (2023). Cardiometabolic Risk Factors Associated with Type 2 Diabetes Mellitus: A Mechanistic Insight. Clin. Med. Insights Endocrinol. Diabetes.

[3] Koskinas K.C., Van Craenenbroeck E.M., Antoniades C., Blüher M., Gorter T.M., Hanssen H., Marx N., McDonagh T.A., Mingrone G., Rosengren A. (2024). Obesity and cardiovascular disease: An ESC clinical consensus statement. Eur. Heart J..

[4] Klein S., Gastaldelli A., Yki-Järvinen H., Scherer P.E. (2022). Why Does Obesity Cause Diabetes?. Cell Metab..

[5] Chandrasekaran P., Weiskirchen R. (2024). The Role of Obesity in Type 2 Diabetes Mellitus—An Overview. Int. J. Mol. Sci..

[6] (2025). World Obesity Federation World Obesity Atlas. https://www.worldobesity.org/resources/resource-library/world-obesity-atlas-2025.

[7] Powell-Wiley T.M., Poirier C.P., Burke V.C.L.E., Després J.-P., Gordon-Larsen P., Lavie C.J., Lear S.A., Ndumele C.E., Neeland I.J., Sanders P. (2021). Obesity and Cardiovascular Disease. Circulation.

[8] He K.-J., Wang H., Xu J., Gong G., Liu X., Guan H. (2024). Global burden of type 2 diabetes mellitus from 1990 to 2021, with projections of prevalence to 2044: A systematic analysis across SDI levels for the global burden of disease study 2021. Front. Endocrinol..

[9] Jin X., Qiu T., Li L., Yu R., Chen X., Li C., Proud C.G., Jiang T. (2023). Pathophysiology of obesity and its associated diseases. Acta Pharm. Sin. B.

[10] Yang M., Liu S., Zhang C. (2022). The Related Metabolic Diseases and Treatments of Obesity. Healthcare.

[11] Wu Y., Li D., Vermund S.H. (2024). Advantages and Limitations of the Body Mass Index (BMI) to Assess Adult Obesity. Int. J. Environ. Res. Public Health.

[12] Sweatt K., Garvey W.T., Martins C. (2024). Strengths and Limitations of BMI in the Diagnosis of Obesity: What is the Path Forward?. Curr. Obes. Rep..

[13] Lin X., Li H. (2021). Obesity: Epidemiology, Pathophysiology, and Therapeutics. Front. Endocrinol..

[14] Lee S.J., Shin S.W. (2017). Mechanisms, Pathophysiology, and Management of Obesity. N. Engl. J. Med..

[15] Bhupathiraju S.N., Hu F.B. (2016). Epidemiology of Obesity and Diabetes and Their Cardiovascular Complications. Circ. Res..

[16] Chung W.K. (2012). An Overview of Mongenic and Syndromic Obesities in Humans. Pediatr. Blood Cancer.

[17] Fitch A.K., Malhotra S., Conroy R. (2024). Differentiating monogenic and syndromic obesities from polygenic obesity: Assessment, diagnosis, and management. Obes. Pillars.

[18] Mahmoud R., Kimonis V., Butler M.G. (2022). Genetics of Obesity in Humans: A Clinical Review. Int. J. Mol. Sci..

[19] Wardle J., Carnell S., Haworth C.M., Plomin R. (2008). Evidence for a strong genetic influence on childhood adiposity despite the force of the obesogenic environment. Am. J. Clin. Nutr..

[20] Jiang L., Penney K.L., Giovannucci E., Kraft P., Wilson K.M. (2018). A genome-wide association study of energy intake and expenditure. PLoS ONE.

[21] Loos R.J.F., Yeo G.S.H. (2022). The genetics of obesity: From discovery to biology. Nat. Rev. Genet..

[22] Ndiaye F.K., Huyvaert M., Ortalli A., Canouil M., Lecoeur C., Verbanck M., Lobbens S., Khamis A., Marselli L., Marchetti P. (2020). The expression of genes in top obesity-associated loci is enriched in insula and substantia nigra brain regions involved in addiction and reward. Int. J. Obes..

[23] Duis J., Butler M.G. (2022). Syndromic and Nonsyndromic Obesity: Underlying Genetic Causes in Humans. Adv. Biol..

[24] Locke A.E., Kahali B., Berndt S.I., Justice A.E., Pers T.H., Day F.R., Powell C., Vedantam S., Buchkovich M.L., Yang J. (2015). Genetic studies of body mass index yield new insights for obesity biology. Nature.

[25] Hardy R., Wills A.K., Wong A., Elks C.E., Wareham N.J., Loos R.J.F., Kuh D., Ong K.K. (2010). Life course variations in the associations between FTO and MC4R gene variants and body size. Hum. Mol. Genet..

[26] Yu K., Li L., Zhang L., Guo L., Wang C. (2020). Association between MC4R rs17782313 genotype and obesity: A meta-analysis. Gene.

[27] Crovesy L., Rosado E.L. (2019). Interaction between genes involved in energy intake regulation and diet in obesity. Nutrition.

[28] Chambers J.C., Elliott P., Zabaneh D., Zhang W., Li Y., Froguel P., Balding D., Scott J., Kooner J.S. (2008). Common genetic variation near MC4R is associated with waist circumference and insulin resistance. Nat. Genet..

[29] Xi B., Chandak G.R., Shen Y., Wang Q., Zhou D. (2012). Association between Common Polymorphism near the MC4R Gene and Obesity Risk: A Systematic Review and Meta-Analysis. PLoS ONE.

[30] Grant S.F.A., Bradfield J.P., Zhang H., Wang K., Kim C.E., Annaiah K., Santa E., Glessner J.T., Thomas K., Garris M. (2009). Investigation of the locus near MC4R with childhood obesity in Americans of European and African ancestry. Obesity.

[31] Sun Y., Sun J., Wu J., Yang M. (2016). Combined effects of FTO rs9939609 and MC4R rs17782313 on elevated nocturnal blood pressure in the Chinese Han population. Cardiovasc. J. Afr..

[32] Tschritter O., Haupt A., Preissl H., Ketterer C., Hennige A.M., Sartorius T., Machicao F., Fritsche A., Häring H.-U. (2011). An Obesity Risk SNP (rs17782313) near the MC4R Gene Is Associated with Cerebrocortical Insulin Resistance in Humans. J. Obes..

[33] Billings L.K., Florez J.C. (2010). The genetics of type 2 diabetes: What have we learned from GWAS?. Ann. N. Y. Acad. Sci..

[34] Aragam K.G., Jiang T., Goel A., Kanoni S., Wolford B.N., Atri D.S., Weeks E.M., Wang M., Hindy G., Zhou W. (2022). Discovery and systematic characterization of risk variants and genes for coronary artery disease in over a million participants. Nat. Genet..

[35] Walsh R., Jurgens S.J., Erdmann J., Bezzina C.R. (2023). Genome-wide association studies of cardiovascular disease. Physiol. Rev..

[36] Ding W., Xu L., Zhang L., Han Z., Jiang Q., Wang Z., Jin S. (2018). Meta-analysis of association between TCF7L2 polymorphism rs7903146 and type 2 diabetes mellitus. BMC Med. Genet..

[37] del Bosque-Plata L., Martínez-Martínez E., Espinoza-Camacho M.Á., Gragnoli C. (2021). The Role of TCF7L2 in Type 2 Diabetes. Diabetes.

[38] Mahley R.W. (2016). Apolipoprotein E: From cardiovascular disease to neurodegenerative disorders. J. Mol. Med..

[39] Toribio-Fernández R., Tristão-Pereira C., Carlos Silla-Castro J., Callejas S., Oliva B., Fernandez-Nueda I., Garcia-Lunar I., Perez-Herreras C., María Ordovás J., Martin P. (2024). Apolipoprotein E-ε2 and Resistance to Atherosclerosis in Midlife: The PESA Observational Study. Circ. Res..

[40] Inouye M., Abraham G., Nelson C.P., Wood A.M., Sweeting M.J., Dudbridge F., Lai F.Y., Kaptoge S., Brozynska M., Wang T. (2018). Genomic Risk Prediction of Coronary Artery Disease in 480,000 Adults: Implications for Primary Prevention. J. Am. Coll. Cardiol..

[41] Abraham G., Havulinna A.S., Bhalala O.G., Byars S.G., De Livera A.M., Yetukuri L., Tikkanen E., Perola M., Schunkert H., Sijbrands E.J. (2016). Genomic prediction of coronary heart disease. Eur. Heart J..

[42] Dudbridge F. (2013). Power and Predictive Accuracy of Polygenic Risk Scores. PLoS Genet..

[43] Zhang M., Ward J., Strawbridge R.J., Celis-Morales C., Pell J.P., Lyall D.M., Ho F.K. (2024). How do lifestyle factors modify the association between genetic predisposition and obesity-related phenotypes? A 4-way decomposition analysis using UK Biobank. BMC Med..

[44] Alexander L., Christensen S.M., Richardson L., Ingersoll A.B., Burridge K., Golden A., Karjoo S., Cortez D., Shelver M., Bays H.E. (2022). Nutrition and physical activity: An Obesity Medicine Association (OMA) Clinical Practice Statement 2022. Obes. Pillars.

[45] Weir C.B., Jan A. (2025). BMI Classification Percentile and Cut Off Points. StatPearls.

[46] Yengo L., Sidorenko J., Kemper K.E., Zheng Z., Wood A.R., Weedon M.N., Frayling T.M., Hirschhorn J., Yang J., Visscher P.M. (2018). Meta-analysis of genome-wide association studies for height and body mass index in ∼700000 individuals of European ancestry. Hum. Mol. Genet..

[47] Mullins V.A., Bresette W., Johnstone L., Hallmark B., Chilton F.H. (2020). Genomics in Personalized Nutrition: Can You “Eat for Your Genes”?. Nutrients.

[48] Singar S., Nagpal R., Arjmandi B.H., Akhavan N.S. (2024). Personalized Nutrition: Tailoring Dietary Recommendations through Genetic Insights. Nutrients.

[49] Nuzzo R.L. (2016). The Box Plots Alternative for Visualizing Quantitative Data. PM&R.

[50] Miranda A.M., Steluti J., Norde M.M., Fisberg R.M., Marchioni D.M. (2019). The association between genetic risk score and blood pressure is modified by coffee consumption: Gene-diet interaction analysis in a population-based study. Clin. Nutr..

[51] Speakman J.R., Loos R.J.F., O’Rahilly S., Hirschhorn J.N., Allison D.B. (2018). GWAS for BMI: A treasure trove of fundamental insights into the genetic basis of obesity. Int. J. Obes..

[52] Frayling T.M., Timpson N.J., Weedon M.N., Zeggini E., Freathy R.M., Lindgren C.M., Perry J.R.B., Elliott K.S., Lango H., Rayner N.W. (2007). A Common Variant in the FTO Gene Is Associated with Body Mass Index and Predisposes to Childhood and Adult Obesity. Science.

[53] Dashti H.S., Miranda N., Cade B.E., Huang T., Redline S., Karlson E.W., Saxena R. (2022). Interaction of obesity polygenic score with lifestyle risk factors in an electronic health record biobank. BMC Med..

[54] Ghatan S., van Rooij J., van Hoek M., Boer C.G., Felix J.F., Kavousi M., Jaddoe V.W., Sijbrands E.J.G., Medina-Gomez C., Rivadeneira F. (2024). Defining type 2 diabetes polygenic risk scores through colocalization and network-based clustering of metabolic trait genetic associations. Genome Med..

[55] Hubacek J.A., Dlouha L., Adamkova V., Dlouha D., Pacal L., Kankova K., Galuska D., Lanska V., Veleba J., Pelikanova T. (2023). Genetic risk score is associated with T2DM and diabetes complications risks. Gene.

[56] Khera A.V., Chaffin M., Wade K.H., Zahid S., Brancale J., Xia R., Distefano M., Senol-Cosar O., Haas M.E., Bick A. (2019). Polygenic Prediction of Weight and Obesity Trajectories from Birth to Adulthood. Cell.

[57] Khera A.V., Chaffin M., Aragam K.G., Haas M.E., Roselli C., Choi S.H., Natarajan P., Lander E.S., Lubitz S.A., Ellinor P.T. (2018). Genome-wide polygenic scores for common diseases identify individuals with risk equivalent to monogenic mutations. Nat. Genet..

[58] Lazarte J., Dron J.S., McIntyre A.D., Skanes A.C., Gula L.J., Tang A.S., Tadros R., Laksman Z.W., Hegele R.A., Roberts J.D. (2021). Role of Common Genetic Variation in Lone Atrial Fibrillation. Circ. Genom. Precis. Med..

[59] Ntalla I., Kanoni S., Zeng L., Giannakopoulou O., Danesh J., Watkins H., Samani N.J., Deloukas P., Schunkert H., UK Biobank CardioMetabolic Consortium CHD Working Group (2019). Genetic Risk Score for Coronary Disease Identifies Predispositions to Cardiovascular and Noncardiovascular Diseases. J. Am. Coll. Cardiol..

[60] Sodero G., Rigante D., Pane L.C., Sessa L., Quarta L., Candelli M., Cipolla C. (2024). Cardiometabolic Risk Assessment in a Cohort of Children and Adolescents Diagnosed with Hyperinsulinemia. Diseases.

[61] Damavandi N., Soleymaniniya A., Bahrami Zadegan S., Samiee Aref M.H., Zeinali S. (2022). Development of a genetic risk score for obesity predisposition evaluation. Mol. Genet. Genom..

[62] Belsky D.W., Moffitt T.E., Sugden K., Williams B., Houts R., McCarthy J., Caspi A. (2013). Development and evaluation of a genetic risk score for obesity. Biodemography Soc. Biol..

[63] Lyall D.M., Celis-Morales C., Ward J., Iliodromiti S., Anderson J.J., Gill J.M.R., Smith D.J., Ntuk U.E., Mackay D.F., Holmes M.V. (2017). Association of Body Mass Index with Cardiometabolic Disease in the UK Biobank: A Mendelian Randomization Study. JAMA Cardiol..

[64] Shah S., Henry A., Roselli C., Lin H., Sveinbjörnsson G., Fatemifar G., Hedman Å.K., Wilk J.B., Morley M.P., Chaffin M.D. (2020). Genome-wide association and Mendelian randomisation analysis provide insights into the pathogenesis of heart failure. Nat. Commun..

[65] Welsh A., Hammad M., Piña I.L., Kulinski J. (2024). Obesity and cardiovascular health. Eur. J. Prev. Cardiol..

[66] Stathias V., Sotiris G.R., Karagiannidis I., Bourikas G., Martinis G., Papazoglou D., Tavridou A., Papanas N., Maltezos E., Theodoridis M. (2012). Exploring genomic structure differences and similarities between the Greek and European HapMap populations: Implications for association studies. Ann. Hum. Genet..

[67] Drineas P., Tsetsos F., Plantinga A., Lazaridis I., Yannaki E., Razou A., Kanaki K., Michalodimitrakis M., Perez-Jimenez F., De Silvestro G. (2019). Genetic history of the population of Crete. Ann. Hum. Genet..

