# Supplementary figures and images for: Genetic Variants in Metabolic Pathways and Their Role in Cardiometabolic Risk: An Observational Study of >4000 Individuals
Source: Biomedicines. 2025 Jul 22;13(8):1791. doi: 10.3390/biomedicines13081791 (PMC12383580; doi:10.3390/biomedicines13081791)

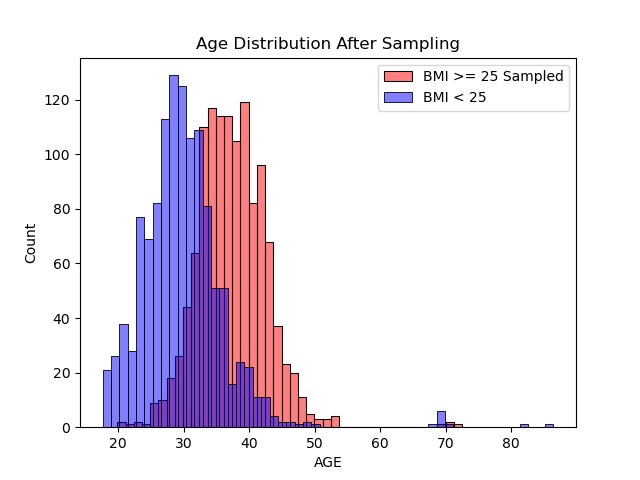

Supplement: Supplementary file 1 [file biomedicines-13-01791-s001.zip › biomedicines-3730122-supplementary.jpeg]
